# Prognostic Value of Red Blood Cell Distribution Width for Patients with Heart Failure: A Systematic Review and Meta-Analysis of Cohort Studies

**DOI:** 10.1371/journal.pone.0104861

**Published:** 2014-08-18

**Authors:** Yuan-Lan Huang, Zhi-De Hu, Shi-Jian Liu, Yi Sun, Qin Qin, Bao-Dong Qin, Wei-Wei Zhang, Jian-Rong Zhang, Ren-Qian Zhong, An-Mei Deng

**Affiliations:** 1 Department of Laboratory Medicine, NO. 455 Hospital of People's Liberation Army, Shanghai, P. R. China; 2 Department of Laboratory Diagnosis, Changhai Hospital, Second Military Medical University, Shanghai, P. R. China; 3 Department of Laboratory Diagnosis, Changzheng Hospital, Second Military Medical University, Shanghai, P. R. China; 4 Department of Laboratory Medicine, General Hospital of Ji'nan Military Region of People's Liberation Army, Ji'nan, Shandong Province, P. R. China; 5 Department of Biobank and Biostatistics, Pediatric Translational Institute, Shanghai Children's Medical Center, School of Medicine, Shanghai Jiaotong University, Shanghai, P. R. China,; Merck & Co., United States of America

## Abstract

**Aims:**

Multiple studies have investigated the prognostic role of red blood cell distribution width (RDW) for patients with heart failure (HF), but the results have been inconsistent. The aim of the present study was to estimate the impact of RDW on the prognosis of HF by performing a systematic review and meta-analysis.

**Methods and Results:**

The Embase, PubMed, and Web of Science databases were searched up to November 16, 2013 to identify eligible cohort studies. The quality of each study was assessed using the Newcastle-Ottawa Scale (NOS). The association between RDW, either on admission or at discharge, and HF outcomes (all-cause mortality [ACM], heart transplantation, cardiovascular mortality, and rehospitalization, etc.) were reviewed. The overall hazard ratio (HR) for the effect of RDW on ACM was pooled using a random-effects model, and the publication bias was evaluated using funnel plots and Eggers' tests. Seventeen studies, with a total of 18288 HF patients, were included for systematic review. All eligible studies indicated that RDW on admission and RDW at discharge, as well as its change during treatment, were of prognostic significance for HF patients. The HR for the effect of a 1% increase in baseline RDW on ACM was 1.10 (95% confidence interval: 1.07–1.13), based on pooling of nine studies that provided related data. However, publication bias was observed among these studies.

**Conclusions:**

HF patients with higher RDW may have poorer prognosis than those with lower RDW. Further studies are needed to explore the potential mechanisms underlying this association.

## Introduction

Heart failure (HF) has been recognized as one of the most severe cardiovascular syndromes worldwide, with a high incidence, prevalence, mortality and morbidity [1]. Accurate risk stratification for the early identification of patients who are at higher risk for poor outcome is critical for the management of HF patients [2], [3]. Laboratory tests, imaging examinations, and clinical signs are three categories of tools widely used in HF prognosis estimation [4], [5]. Among these, laboratory tests are of great interest because the results are objective [6], [7]. By contrast, clinical signs and imaging examinations are subjective in nature, and the accuracy of these measures is greatly affected by the experience of the clinicians.

Red blood cell distribution width (RDW) is a routine parameter of hematologic tests that is used to measure the variation in circulating erythrocyte volume [8]. For a long time, RDW has been regarded as a useful index to differentiate the etiology of anemia, such as thalassemia and megaloblastic anemia, as well as iron deficiency-related anemia [9]. Recently, the clinical significance of RDW in non-hematologic disorders, such as liver diseases [10], [11], autoimmune diseases [12], [13], respiratory diseases [14], [15], stroke [16], [17], critical illness [18], [19], and cardiovascular diseases [20], has been extensively investigated. To date, many studies have explored the prognostic value of RDW for HF, but the results have been inconsistent. Meta-analysis, a statistical method to integrate the findings of available studies, has recently been recognized as an effective strategy to draw a robust and reliable conclusion on a certain topic. Therefore, we performed a systematic review and meta-analysis to estimate the prognostic value of RDW for patients with HF.

## Materials and Methods

### Literature searching

Two authors (ZD Hu and YL Huang) independently searched the PubMed, Embase, and Web of Science databases to identify eligible studies published up to November 16, 2013. The search terms for the Pubmed search were: “heart failure” OR “cardiac failure” AND “RDW” OR “red cell distribution width” OR “erythrocyte indices”. A similar search strategy was used for searching Embase. Manual searches were also performed by reviewing the references of the eligible studies and reviews on this topic.

### Inclusion criteria

Studies that fulfilled the following inclusion criteria were included: 1) cohort studies that evaluated the prognostic value of RDW for patients with HF, either acute or chronic HF; 2) studies with a follow-up duration of more than 1 year; 3) studies that reported at least one of the following outcomes: all-cause hospitalization, HF-related hospitalization, cardiovascular death, all-cause mortality (ACM), and heart transplantation. Studies that met any of the following exclusion criteria were excluded: 1) animal or cell line studies; 2) duplicated publications; 3) conference abstracts; and 4) manuscripts published in languages other than English. Two authors (ZD Hu and YL Huang) independently reviewed the abstracts and titles of the retrieved studies to identify potentially eligible studies. If necessary, review of the complete text was performed. Disagreements were resolved by discussion and consensus.

### Data extraction

Data extraction and quality assessment were performed independently by two authors (ZD Hu and YL Huang). The following data were extracted from eligible studies: names of the first authors, publication year, sources of participants, sample sizes, participants' characteristics, follow-up durations, event rates, endpoints with their corresponding hazard ratios (HRs) and 95% confidence intervals (CIs), and the confounding factors adjusted for. The corresponding authors of the eligible studies were not contacted for detailed information if the necessary data were not reported in the full-text of the papers.

The Newcastle-Ottawa Scale (NOS) [21], with minor modifications, was used to assess the quality of the included studies. This quality assessment tool consists of three domains, including selection of the exposed and unexposed cohort (maximum: four stars), comparability of the two cohorts (maximum: two stars), and outcome assessment (maximum: three stars). Two authors (ZD Hu and YL Huang) independently performed quality assessment, and any disagreements were resolved by discussion with a third author (SJ Liu, AM Deng, or RQ Zhong) who was blinded to the previous results.

### Statistical analysis

This meta-analysis was performed and reported in accordance with the PRISMA guidelines for systematic reviews and meta-analyses (Checklist S1) [22]. The most fully-adjusted HR reported in the original articles was extracted. For example, in a study in which the unadjusted and multivariable adjusted HRs (95% CIs) were 2 (1.8–2.3) and 1.5 (1.3–1.8), the latter HR was extracted. Given that the endpoints, as well as the value assignment strategy for RDW across eligible studies, were variable, and it was not reasonable to pool HRs derived from different endpoints, only the studies that provided an HR for an increment of 1% unit RDW for ACM in HF patients were included in the meta-analysis. Cochrane's Q test (significance level of P<0.10), as well as the *I^2^* statistics, were used to assess the heterogeneity among the included studies. If *I^2^*>25% or *P*<0.10, we pooled the reported HRs using a random-effects model; otherwise, the HRs were pooled using a fixed-effects model. To explore the possible source of heterogeneity, subgroup analysis was performed according to the origins of participants (USA or Europe), follow-up duration (median or mean follow-up time >2 years or ≤2 years), study design (prospective, post-hoc, or retrospective), and whether natriuretic peptides (B-type natriuretic peptide [BNP] or N-terminal B-type natriuretic peptide [NT-proBNP]) were adjusted for in calculating the adjusted HRs. Sensitivity analysis was conducted to determine whether the exclusion of any single study would result in a significant change in the final results. Funnel plots and the Egger's test were applied to evaluate the potential publication bias [23]. All analyses were performed using STATA 12.0 (Stata Corp LP, College Station, TX).

## Results

### Summary of eligible studies

**Figure 1:**
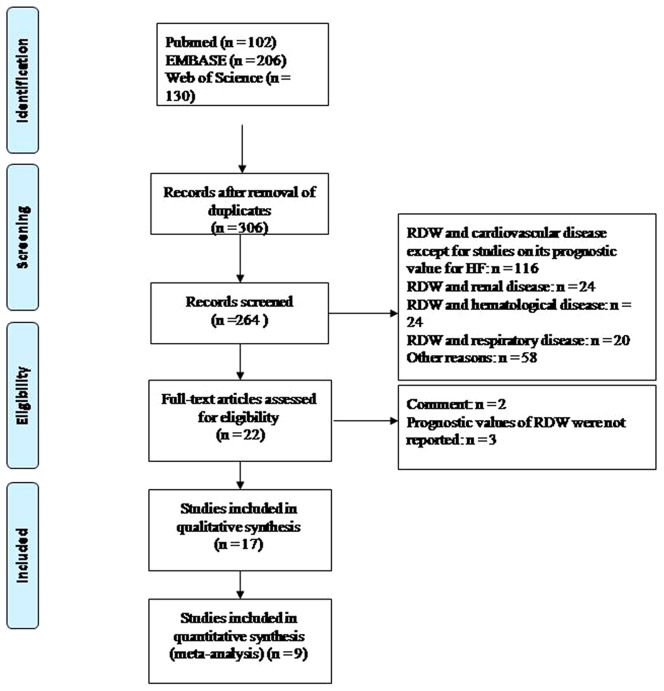
Flow chart for study identification and inclusion.

A flowchart outlining our literature search is shown in **Figure 1**. Overall, 17 cohort studies, with a total of 18288 HF patients, were included in our systematic review [24]–[40]. The study performed by Felker*et et al* [29] analyzed the prognostic value of RDW in two independent cohorts (CHARM and Duke Databank). Therefore, it was regarded as two independent cohort studies. A summary of the characteristics of the eligible studies is given in **Table 1**. Nine [24], [25], [27], [30]–[33], [35], [39] of the included studies were prospective cohort studies, whereas four [26], [28], [34], [38] were retrospective studies and the other four [29], [36], [37], [40] were post hoc analyses.

**Table 1:** Summary of eligible studies.

First Author	Country	Year	Sample size	Study design	Participants	Follow-up duration (years)	Quality assessment by NOS		
							Selection	Comparability	Outcome
Allen [24]	USA	2010	1012	Prospective	HF>18 years	1.0±0.3	★★★★	★★	★★★
Al-Najjar [25]	UK	2009	1087	Prospective	HF with LVEF <45%	4.33 (IQR: 2.41–5.48)	★★★★	★	★★
Aung [26]	UK	2013	274	Retrospective	HF with LVEF <45%	2.25 (IQR: 1.5–3.33)	★★★	★	★★★
Bonaque [27]	Spain	2012	698	Prospective	Outpatients with chronic HF	2.5 (IQR:1.2–3.7)	★★★★	★★	★★★
Cauthen [28]	USA	2012	6052	Retrospective	Outpatients with chronic HF	4.4±2.4	★★★	★★	★★★
Felker [29], CHARM	USA	2007	2679	Post hoc analysis	HF	Median: 2.83	★★★	★★	★★★
Felker [29], Duke Databank	USA	2007	2140	Post hoc analysis	HF undergoing CRT	Median: 4	★★★	★★	★★★
Forhecz [30]	Hungary	2009	195	Prospective	HF with LVEF <45%	1.21 (range: 0.08–2.08)	★★★★	★	★★★
Jackson [31]	UK	2009	707	Prospective	HF more than 18 years	1.15 (range: 0.57–2.17)	★★★★	★	★★
Jung [32]	Germany	2011	354	Prospective	Chronic HF	6.66±0.96	★★★★	★	★★★
Makhoul [33]	Israel	2013	614	Prospective	Acute decompensated HF	1	★★★	★★	★★
Oh [34]	Korea	2012	261	Retrospective	Acute decompensated HF	1.74 (IQR: 0.62–2.50)	★★★	★	★★★
Pascual-Figal [35]	Spain	2009	628	Prospective	Acute HF	3.12 (IQR: 1.38–4.09)	★★★	★★	★★
Rickard [36]	USA	2012	217	Post hoc analysis	HF undergoing CRT	4.4±1.8	★★★	★	★★★
van Kimmenade [37]	USA	2009	205	Post hoc analysis	Acute HF	1	★★★	★	★★★
Zalawadiya [38]	USA	2011	789	Retrospective	Decompensated HF	Median: 1.57	★★★	★★	★★★
Holmstrom [39]	Sweden	2013	179	Prospective	HF with LVEF <50%	2.00±0.58	★★★★	★	★★
Simbaqueba [40]	USA	2013	197	Post hoc analysis	Chronic systolic HF	5	★★★	★	★★★

CRT: cardiac resynchronization therapy, HF: heart failure, IQR: interquartile range, LVEF: left ventricular ejection fraction, NOS: Newcastle-Ottawa Scale (NOS).

For quality assessment, eight of the studies were considered as having mild cohort selection bias because they were designed as retrospective studies [26], [28], [34], [38] or post hoc analyses [29], [36], [37], [40]. Ten eligible studies [25], [26], [30]–[32], [34], [36], [37], [39], [40] were regarded as having moderate comparability, because confounding factors were not fully adjusted for, particularly some of the well recognized prognostic factors for HF, including the New York Heart Association (NYHA) functional classification, natriuretic peptide levels, left ventricular ejection fraction (LVEF), and cardiac troponin levels. The four prospective studies [25], [31], [35], [39] were regarded as having mild outcome bias, because they did not report the censors.

### Main findings of eligible studies

**Table 2** lists the main findings of all the eligible studies. The association between RDW on admission and ACM was investigated in 12 studies [24]–[31], [35]–[37], [39]. Of these studies, two reported the HRs for a standard deviation (SD) increment in RDW for ACM in HF [29], [30], and the rest reported the HRs for a 1% increment in RDW. Three studies investigated the impact of RDW changes on ACM [26], [28], [33], and two studies investigated of the effects of RDW at discharge [35], [38]. The effect of RDW on all-cause hospitalization [24], HF-related hospitalization [27], cardiovascular death or HF-related hospitalization [29], [34], ACM or HF-related hospitalization [30], ACM and heart transplantation [32], and the 1-year mortality or readmission for HF [33] were also investigated in some of the included studies. The confounding factors adjusted for among the eligible studies varied, as shown in **Table 2**, and some well-recognized confounding factors, such as age (n = 16), renal function (n = 14), hemoglobin (n = 12), NYHA class (n = 7), LEVF (n = 5), serum natriuretic peptides (n = 6), and troponins (n = 2), were taken into consideration. All of the studies found that RDW, either on admission, at discharge, or in terms of changes during treatment, was an effective prognostic factor for patients with HF.

**Table 2:** Main findings of the eligible studies.

Author	Endpoints and corresponding HRs	Adjusted factors
Allen LA [24]	ACM:1.07 per 1% All-cause hospitalization: 1.05 per 1%	Age; Hb; NYHA; IHD; Hypertension; LVEF; eGFR; SBP; DBP; ATF; edema; diuretic; loop diuretic
Al-Najjar Y [25]	ACM: 1.12 per 1%	Age; NT-proBNP; WBC; Na; BUN
Aung N [26]	ACM: 1.13 per 1% for baseline and 4.40 for changes in RDW	Cre; Hb; Na; albumin; NYHA
Bonaque JC [27]	ACM: 1.15 per 1% Admission for HF: 1.13 per 1%	Age; hypertension; NYHA; COPD; ATF; Hb; eGFR; gender; DM; LVEF; IA; previous stroke; beta-blocker
Cauthen CA [28]	ACM: 1.09 per 1% at baseline and 1.21× change in RDW	Hb; age; BNP; LDL cholesterol; eGFR LVEF; diastolic stage left atrial area
Felker GM [29], CHARM	Cardiovascular death or HF hospitalization: 1.17 per SD ACM: 1.12 per SD	Bilirubin total; lymphocytes; UA; HbA1c; Hb; CRE; phosphorus inorganic; age; ejection fraction; DM (insulin treated); DM; prior HF hospitalization within 6 months; prior HF hospitalization but not within 6 months; cardiomegaly; NYHA; HF history; BBB; randomization to candesartan
Felker GM [29], Duke Databank	ACM: 1.29 per SD	Age; Hb; number of diseased vessels; noncardiac Charlson index; SBP; ejection fraction; hypertension; gender
Forhecz Z [30]	ACM: 1.61 per SD ACM or CHF hospitalization: 1.29 per SD	Age; eGFR; Hb; BMI; DBP; Na; NT-proBNP
Jackson CE [31]	ACM: 1.06 per 1%	BNP; age; WBC; gender; Hb; lymphocyte
Jung C [32]	ACM or heart transplantation: 1.53 per 1%	Age, gender, BMI, NYHA, Hb; CRP, ESR
Makhoul BF [33]	The 1-year mortality or readmission for HF: 1.15 per 1% at baseline and 1.23× change in RDW	Age, gender, DM, hypertension, smoking, eGFR, BUN, Na, ATF, troponin I, BNP; medical therapy
Oh J [34]	CV mortality or rehospitalization: 1.14× at baseline and 1.74× change in RDW per 1%	HF history; IA; ATF; ACEI/ARB; age; SBP; cholesterol; UA; eGFR; Hb
Pascual-Figal DA [35]	ACM: 1.07 (RDW at discharge) per 1%	Hb; age; NYHA; HF history; β-blockers; eGFR; prior stroke; BBB; gender; hypertension; COPD; ATF; IA; LVEF; LV end-diastolic diameter; left atrial diameter; CRP; cholesterol
Rickard J [36]	ACM: 1.19 per 1%	Gender, eGFR; NSAVCD
van Kimmenade RR [37]	ACM: 1.03 per 1%	NT-proBNP; ST2; BUN; age; murmur, SBP
Zalawadiya SK [38]	ACM: 1.17 (RDW at discharge) per 1%	Age, BMI, SBP, HR, DM,PAB, hypothyroidism, family history of CAD, statin therapy, aspirin use, CRE, Hb; MCV
Holmstrom A [39]	ACM: 2.04 per 1%	Pulmonary artery pressure, DM, eGFR, highly sensitive troponin T
Simbaqueba C [40]	ACM, heart transplant or HF-related hospitalization: 1.34 per 0.133% units	Age, LVEF, eGFR, NYHA, mean corpuscular hemoglobin concentration

HF: heart failure, BMI: body mass index; CRP: C-reactive protein; CAD: coronary heart disease; SBP: systolic blood pressure; HF: heart rate; DM: diabetes mellitus; Hb: hemoglobin; MCV: mean corpuscular volume; CRE: creatinine; BUN: blood urea nitrogen; ESR: erythrocyte sedimentation rate; NYHA: New York Heart Association functional classification; WBC: white blood cells; ATF: atrial fibrillation; Na: serum sodium; BBB: bundle branch block PAB: peripheral arterial disease; ACM: All-cause mortality; IHD: ischemic heart disease; DBP: diastolic blood pressure; CRT: cardiac resynchronization therapy; COPD: chronic obstructive pulmonary disease; IA: ischemic etiology; ACEI: angiotensin-converting enzyme inhibitors (ACEIs); ARB: angiotensin receptor blockers; eGFR: estimated glomerular filtration rate; IHD: ischemic heart disease; CV: cardiovascular; UA: uric acid; NSAVCD: non-specific intraventricular conduction delay.

### RDW and HF outcomes

**Figure 2:**
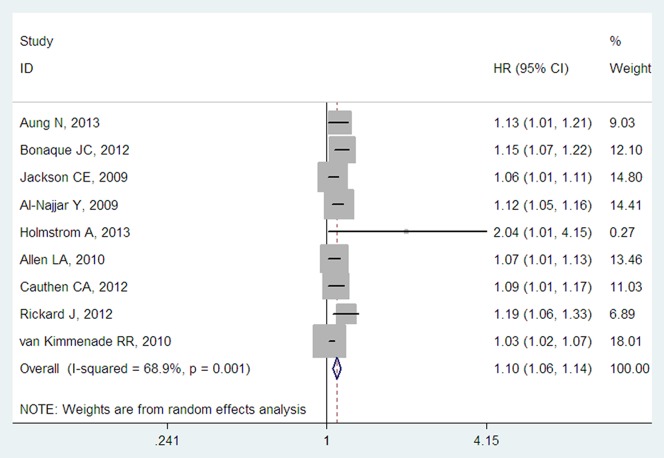
Forest plot of the HR for each 1% increase in RDW for ACM in HF patients. Each solid rectangle represents an eligible study. The size of each solid rectangle reflects the sample size of the eligible study. Error bars represent 95% confidence intervals (CIs). RDW, red blood cell distribution width; HF, heart failure.

Nine studies reported the adjusted HRs for a 1% increase in RDW on admission for ACM [24]–[28], [31], [36], [37], [39] in HF patients, and therefore, it was reasonable to include these studies in the meta-analysis. The pooled HR, based on the HRs from the abovementioned nine studies, showed that for patients with HF, each 1% increase in RDW on admission was associated with a 10% higher risk of future mortality events (adjusted HR per 1% RDW: 1.10, 95% CI: 1.06–1.14, **Figure 2**). The *I^2^* was 68.9% (*P*<0.01), suggesting that considerable heterogeneity existed among the eligible studies.

### Subgroup analysis and sensitivity analysis

To explore possible sources of heterogeneity among the eligible studies, subgroup analysis was performed. As shown in **Table 3**, study design, study location, and whether natriuretic peptide was adjusted for seemed to have no apparent effect on pooled HRs, because the heterogeneity remained in the subgroups according to the above characteristics. However, we found that the follow-up duration in the included studies had a significant impact on the pooled HR, and the pooled HR was higher for studies with a follow-up duration exceeding 2 years. More importantly, heterogeneity was resolved in subgroup analysis, indicating that the duration of follow-up was one of the sources of heterogeneity among the included studies.

**Table 3:** Subgroup analysis.

	N	Model	Meta-analysis		Heterogeneity	
			Pooled HR	95% Confidence interval	I^2^ (%)	P
**Data collection**						
Prospective	5	Random	1.10	1.05–1.15	52.3	0.079
Retrospective or post hoc	4	Random	1.09	1.02–1.17	71.6	0.014
**Locations of study**						
USA	4	Random	1.07	1.02–1.13	64.2	0.039
Europe	5	Random	1.11	1.06–1.16	48.8	0.099
**Follow-up duration**						
>2 years	5	Fixed	1.13	1.09–1.16	0.0	0.717
≤2 years	4	Fixed	1.04	1.02–1.06	47.6	0.126
**Natriuretic peptide adjusted**						
No	5	Fixed	1.12	1.08–1.16	43.3	0.133
Yes	4	Random	1.07	1.03–1.11	70.2	0.018

**Figure 3:**
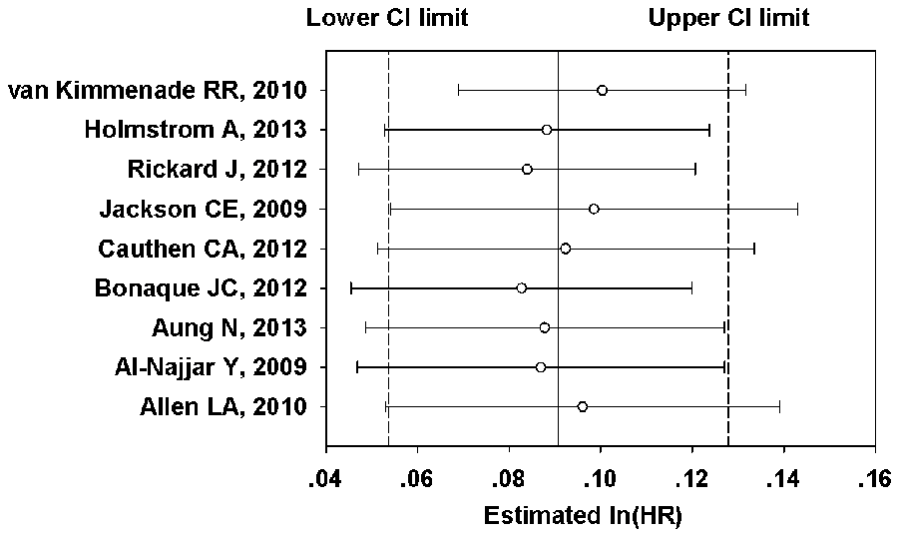
Sensitivity analysis for the association between RDW and ACM in HF patients. RDW, red blood cell distribution width; HF, heart failure; ACM, all-cause mortality.

The results of a sensitivity analysis showed that omission of each study individually did not significantly influence the overall results (**Figure 3**), indicating that the results of the present meta-analysis are stable.

### Publication bias

**Figure 4:**
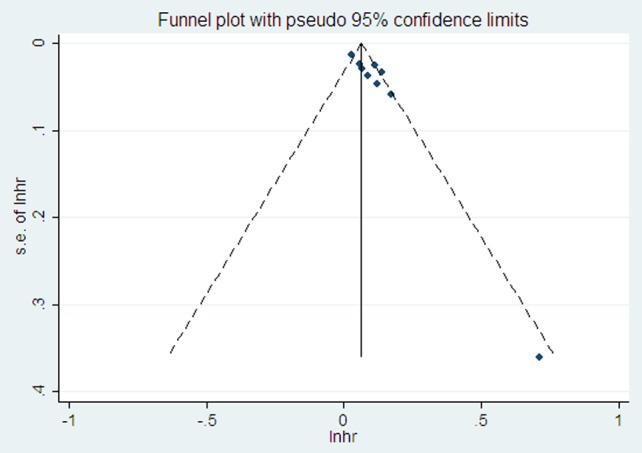
Funnel plots for the assessment of potential publication bias in studies used to analyze RDW for predicting ACM in HF patients. Each solid rectangle represents an eligible study in the meta-analysis. The center line indicates the summarized HR. RDW, red blood cell distribution width; HF, heart failure; ACM, all-cause mortality.

**Figure 4** shows a funnel plot for the nine studies that were included in the meta-analysis, and obvious asymmetry was observed. The results of Egger's test gave a co-efficient of bias of 2.78 (*P* = 0.002), also indicating that potential publication bias existed across the eligible studies.

## Discussion

The present systematic review and meta-analysis indicated that increased RDW, either at baseline or at discharge, as well as the change in RDW during treatment are associated with poor prognosis in patients with HF. The meta-analysis of the results of nine studies demonstrated that the pooled HR for a 1% increase in RDW for ACM was 1.10, suggesting that the risk of ACM increases by 10% with each 1% increase in the baseline RDW. Prognostic evaluation is especially important for HF management, because it will greatly affect clinical decisions. Our results indicate that RDW is an effective index for HF prognosis evaluation. This means RDW should be measured when comprehensively assessing the prognosis of HF patients, and more intensive treatment for HF may be needed for patients with a higher RDW.

The exact mechanisms underlying the association between RDW and poor prognosis for patients with HF remain unknown at this stage. One suggested hypothesis is that inflammation may bridge the relationship between higher RDW and poorer HF prognosis. It is well documented that the inflammatory response plays a critical role in the development and progression of HF [41], [42]. Inflammatory biomarkers, as indicated by previous evidence, can provide important prognostic information for HF [43]. On the other hand, it is widely accepted that inflammation inhibits erythrocyte maturation and accelerates the migration of reticulocytes into the peripheral circulation, thereby increasing RDW [44]–[46]. Indeed, the positive relationships between RDW and inflammatory indices have also been documented [30], [47], [48]. Our previous studies also showed that glucocorticoid, a well-known anti-inflammatory agent, could reduce the RDW in patients with systemic lupus erythematosus [13]. Together, these findings indicate that inflammation plays an important role in the association between a relatively higher RDW and poor HF prognosis. Further studies are needed to explore the detailed mechanisms of the relationships between RDW and HF prognosis.

Compared with traditional prognostic indices, such as BNP, NT-proBNP, midregional pro-atrial natriuretic peptide (MR-proANP), and troponins [6], [7], RDW as a prognostic factor for patients with HF offers at least three advantages. First, it is an inexpensive index. Because blood cell count is a routine test for patients with HF and RDW is a regular hematologic parameter, no additional cost should be needed to introduce RDW into the estimation of HF prognosis. Second, RDW is an easily acquired index, which can be tested even in a community hospital. Third, the lifespan of red blood cells is approximately 130 days [49], which is much longer than that of natriuretic peptides [50], [51]. Therefore, RDW may have less biological variation, and this characteristic may make its clinical interpretation much easier than the parameters evaluated in traditional HF laboratory tests.

The results of our subgroup analyses suggest that follow-up duration is an important source of heterogeneity among the included studies, and the association between a higher RDW and a higher risk for future ACM events seemed to be stronger in studies with longer follow-up durations (>2 years). More importantly, the heterogeneity was resolved in subgroup analysis according to follow-up duration. These results indicate that the prognostic value of RDW may be underestimated by studies with shorter follow-up durations. It should be noted that although adjustment for natriuretic peptides was not the source of heterogeneity, it had a moderate effect on pooled HRs. This result indicates that the prognostic value of RDW might be overestimated by studies with no adjustment for BNP or NT-proBNP. In addition, sensitivity analysis was performed to explore the impact of each individual study on the overall outcomes of the meta-analysis. The results showed that no individual study had an obvious effect on the pooled HR, suggesting that the results of the present meta-analysis are stable.

The present systematic review and meta-analysis has some limitations. First, only studies published in English were included; therefore, study selection bias could not be completely excluded. Second, only nine studies were included in the final meta-analysis, and publication bias was observed. Therefore, the prognostic value of RDW for HF may be overestimated. Third, the confounding factors adjusted in individual studies varied; some well-established indices, including natriuretic peptides, troponins, renal function, and history of cardiovascular events, were not fully adjusted for in some of the studies. Inadequate adjustment for confounders may lead to bias, either towards over- or under-estimation of the HR of RDW. Residual or unknown confounding factors cannot be excluded as possible interpretations for the observed findings, as well as heterogeneity across eligible studies. Further studies, with larger sample sizes, longer follow-up durations, various defined endpoints, and adequate adjustments for confounding factors, should be performed to rigorously confirm the prognostic value of RDW for patients with HF.

In conclusion, the results of the present systematic review and meta-analysis indicate that RDW, an easily and inexpensively acquired index, is of prognostic significance for patients with HF. Further studies are needed to uncover the potential mechanisms underlying the associations between a higher RDW and poor prognosis in HF patients. However, the potential prognostic value of RDW should be taken into consideration in the comprehensive management of patients with HF.

## Supporting Information

Checklist S1 **PRISMA Checklist.**
(DOC)
